# Evaluating contribution of the cellular and humoral immune responses to the control of shedding of *Mycobacterium avium* spp. *paratuberculosis* in cattle

**DOI:** 10.1186/s13567-015-0204-1

**Published:** 2015-06-19

**Authors:** Vitaly V. Ganusov, Don Klinkenberg, Douwe Bakker, Ad P. Koets

**Affiliations:** Department of Microbiology, University of Tennessee, Knoxville, TN 37996 USA; Centre for Infectious Disease Control, National Institute for Public Health and the Environment, Bilthoven, The Netherlands; Department of Bacteriology and TSE, Central Veterinary Institute part of Wageningen UR, Lelystad, The Netherlands

## Abstract

*Mycobacterium avium* spp. *paratuberculosis* (MAP) causes a persistent infection and chronic inflammation of the gut in ruminants leading to bacterial shedding in feces in many infected animals. Although there are often strong MAP-specific immune responses in infected animals, immunological correlates of protection against progression to disease remain poorly defined. Analysis of cross-sectional data has suggested that the cellular immune response observed early in infection is effective at containing bacterial growth and shedding, in contrast to humoral immune responses. In this study, 20 MAP-infected calves were followed for nearly 5 years during which MAP shedding, antigen-specific cellular (LPT) and humoral (ELISA) immune responses were measured. We found that MAP-specific cellular immune response developed slowly, with the peak of the immune response occurring one year post infection. MAP-specific humoral immunity expanded only in a subset of animals. Only in a subset of animals there was a statistically significant negative correlation between the amount of MAP shedding and magnitude of the MAP-specific cellular immune response. Direct fitting of simple mechanistic mathematical models to the shedding data suggested that MAP-specific immune responses contributed significantly to the kinetics of MAP shedding in most animals. However, whereas the MAP-specific cellular immune response was predicted to suppress shedding in some animals, in other animals it was predicted to increase shedding. In contrast, MAP-specific humoral response was always predicted to increase shedding. Our results illustrate the use of mathematical methods to understand relationships between mycobacteria and immunity in vivo but also highlight problems with establishing cause-effect links from observational data.

**Electronic supplementary material:**

The online version of this article (doi:10.1186/s13567-015-0204-1) contains supplementary material, which is available to authorized users.

## Introduction

Mycobacterial infections represent major health problems both in humans and in farm animals [1,2]. *Mycobacterium avium* subsp. *paratuberculosis* (MAP) is the causative agent of Johne's disease in ruminants such as cows and sheep [3] causing chronic inflammation of the small intestine. Exposed animals enter a subclinical period of 2 to 5 years after which a proportion of the infected animals develops a severe enteropathy with chronic diarrhea and ultimately death [4]. In cattle, calves predominantly acquire the infection in the first months of life via MAP-contaminated milk or grass [3]. After crossing the epithelial barrier through Peyer’s patches, MAP is phagocytized by macrophages. Inside macrophages, bacteria interfere with phagosome maturation leading to MAP replication [4,5]. MAP multiplies slowly until it kills the macrophage, which bursts and releases the bacteria. Killing of macrophages and associated inflammatory reactions attract more macrophages to the site of infection, which in turn get infected and subsequently killed [5,6]. This process of MAP replication and macrophage killing results in the formation of granulomas containing macrophages with high intracellular bacterial burden [7]. MAP is shed into the lumen of the gut and excreted with feces but the exact mechanisms by which MAP exits granulomas and is secreted into the gut lumen are not well understood. Low level shedding may occur within several weeks post infection, followed by a latent phase. After the latent phase, the length of which varies greatly between cows, the animals progress into a phase of real or apparent intermittent fecal shedding but as the disease develops, shedding in feces becomes continuous [8].

There is a licensed vaccine against Johne’s disease, Mycopar®, which contains inactivated MAP with an oil adjuvant [9]. While the vaccine has some side effects its impact on the prevention of the infection and disease are inconclusive with some studies showing protection against infection but no impact on disease progression while others showed protection against Johne’s disease [6,9,10]. Mycopar® induces both MAP-specific cellular (CD4 T cells) and humoral (antibodies) responses but the correlates of protection against the infection or disease have not been clearly defined [6]. Understanding which arms of the immune response control bacterial replication will be instrumental for the development of more efficacious vaccines.

Previous work measuring adaptive immune responses in MAP-infected cows found an early cellular immune response (aimed at killing intracellular MAP in macrophages) followed by a late humoral response (aimed at removing extracellular bacteria) during the course of the disease [11]. MAP-specific cellular immune response is characterized by the production of IFN-γ [11,12] which activates macrophages to kill intracellular MAP [3]. The cellular immune response is often unable to completely eliminate the bacteria leading to the establishment of a steady state between MAP and immunity (the latent/subclinical phase). Late in infection, bacterial shedding in feces increases, which coincides with the decline in IFN-γ-producing cells, increase in IL-10-producing cells, and rise of MAP-specific antibodies [3,11].

**Figure 1:**
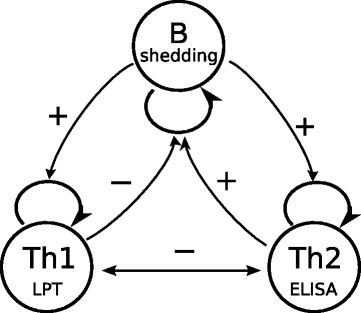
**Relationships between cellular and humoral immune responses specific to Mycobacterium avium spp. paratuberculosis (MAP) and MAP shedding.** From the basics of immunology we expect that presence of bacteria will stimulate MAP-specific immune responses. While MAP-specific immune responses are expected to impact bacterial replication, whether these immune responses reduce or enhance shedding is not well understood. It is believed that cellular (Th1) immune response suppresses bacterial replication, while humoral (Th2) immune response enhances bacterial replication. Furthermore, the relationship between cellular and humoral immunity (co-expression or cross-suppression) remains incompletely defined although it is generally thought that the two responses are cross-inhibitory [13].

Collectively, the data from these studies have been used to argue that chronic progressive forms of paratuberculosis involve a switch in the host immune response according to the murine Th1-Th2 paradigm [13]. According to this paradigm (Figure 1), in analogy with human tuberculosis, MAP can induce both types of the immune response, but early during infection the cellular (Th1) response dominates, which leads to inhibition of the humoral (Th2) response, effective control of MAP replication, and limited bacterial shedding. Later during the infection, the cellular response is replaced by the humoral response, which inhibits the cellular response and is much less effective against MAP [14–16].

Most of the data leading to these conclusions have been generated using cross-sectional studies, and establishing a cause-and-effect relationship from such studies is difficult [17]. For instance, it remains undefined if MAP-specific antibodies play a significant role in control of bacterial shedding or in fact speed up disease progression by increasing the rate of uptake of bacteria by macrophages [18]. Recent data in sheep indicated that both higher early IFN-γ and higher early IL-10 are associated with delayed shedding [19], whereas other studies indicate that both cellular and humoral responses may be impaired during later stages of disease [7]. These data are in conflict with the Th1-Th2 paradigm and suggest that the loss of cellular immunity and appearance of antibodies may be simply indicative of disease progression, rather than resulting from direct competition between two branches of the adaptive immunity as has been argued recently [20,21].

Experimental infection of animals is a powerful method to investigate factors that contribute to the rate at which infected animals progress to disease [12]. In experiments, the timing of infection and the dose are known precisely, and animals can be monitored longitudinally with measurements of the MAP-specific immune responses and bacterial shedding. Here we present infection experiments of MAP-infected cows followed up to five years, and use statistical analyses and mathematical modeling to investigate whether and how cellular and humoral immune responses control shedding in these experimentally infected calves.

## Materials and methods

### Animals

This experiment has these experiments have been previously described in detail in a PhD thesis [22]. Twenty Holstein-Friesian calves were purchased at birth from different commercial farms and housed at the specific pathogen free (SPF) facilities of the Central Veterinary Institute (CVI) in Lelystad, The Netherlands, throughout the experimental period. Animals were kept on a regular feed regimen according to their age and lactation status but never received fresh grass. Experimental procedures were approved by the Ethical Committee of the CVI. Calves were followed over an experimental period of 55 months (from 31-01-1999 to 27-08-2003). During the course of the investigation period only 7 out of the initial 20 cattle survived to the end. Post-mortem examinations of animals that died during the study showed a diverse number of causes of death, none of which were directly due to the experimental infection with MAP. This experiment was designed to run with conditions as closely to common Dutch dairy farming practice as possible. Therefore, all animals were bred at 15 months of age in order for them to calve and to start milk production at about 2 years of age. A major cause for animals to be culled was infertility (*n* = 6). Cows that did not conceive were culled at about 2 years of age. Two animals were culled early following the first calving. One was culled due to severe lameness, the other due to fatty liver syndrome. The remaining 5 animals were culled during the last 6 months of the study due to common disorders such as lameness and mastitis. None of the animals developed any signs of clinical paratuberculosis (severe diarrhea, weight loss, emaciation, edema). In Dutch dairy herds the average life span of a cow is just over 4 years and losses during the current study did not exceed losses as observed on well managed commercial dairy farms.

### Experimental infection

Calves were infected with 20 grams of MAP contaminated feces given orally, three times a week for a period of four weeks during the first month of life. The inoculum was obtained from a cow with clinical signs of MAP infection consistently shedding IS900-positive MAP. The time “0” in our experimental data denotes the day the first blood and fecal samples were taken from the calf, just prior to the first dose of oral MAP infection.

### Fecal shedding measurements

Rectal samples for fecal culture were taken as previously described [22], approximately every two weeks. Bacteria were cultured according to a modified method of Jorgenson [23]. Growth of MAP was mycobactin dependent and was checked every 4 weeks. If no growth was observed after 6 months of culture, the sample was considered negative. The presence of MAP in positive cultures was confirmed by amplification of the MAP specific IS900 by PCR [24]. Shedding data was expressed semi-quantitatively in 4 categories: 0 = negative, 1 or “+” =1-10 cfu/slant, 2 or “++” = 11-100 cfu/slant, 3 or “+++” > 100 cfu/slant.

### Blood sampling

Blood was collected from the jugular vein into heparinized tubes and into serum tubes (BD Vacutainer, Becton, Dickinson, Europe), in approximately one-month intervals. Heparinized blood was used for the isolation of peripheral blood mononuclear cells (PBMC). Serum was stored at −20 °C and processed at a later time-point.

### Antigen

Purified protein derivative (PPD-P, Johnine) antigen was used in the Lymphocyte Proliferation Test and the ELISA. PPD-P was produced at CVI, Lelystad, as previously described, from the MAP strains 3 + 5 and C [25].

### Cellular immune response measurements

Peripheral blood mononuclear cells (PBMC) were isolated and cultured according to the methods described in detail elsewhere [15]. Lymphocyte Proliferation Tests (LPT) were performed according to the methods described in detail previously [15]. In short, cells were cultured in 96-well microtitre plates using 100 μL of the PBMC suspension and 100 μL of antigen per well in triplicates. The purified PPD-P antigen was used in predetermined optimal concentrations of 10 μg/mL. Concanavalin A (ConA) was used as a positive control (2.5 μg/mL) and medium alone as a negative control. Cells were cultured at 37 °C and 5% CO_2_ in a humidified incubator for 3 days. Then 0.4 μCi (=14.8 × 10^3^ Bq) ^3^H (tritiated) thymidine (Amersham International) was added to each well and cells were cultured for an additional 18 h. Subsequently, cells were harvested onto glass fiber filters. Incorporation of ^3^H thymidine was measured by liquid scintillation counting, and expressed as counts per minute (cpm). Cpm is used as a measure for the intensity of the MAP-specific cellular Th1 response and was denoted as LPT.

### Humoral immune response measurements

Antibodies (total IgG) specific for PPD-P were detected by ELISA according to the method described earlier [26]. All sera were 10 × diluted in blocking buffer. Results were expressed as background corrected mean optical densities, measured at 405 nm wavelength (OD405nm). OD405nm was used as a measure for the intensity of the MAP-specific humoral (Th2) immune response and was denoted as ELISA.

### Statistical analyses

We used three alternative statistical approaches to investigate associations between cellular and humoral immune responses and MAP shedding, and used all results together to understand how MAP-specific immunity contributes to disease progression in MAP-infected animals. The first approach was to calculate correlations between kinetics of MAP shedding and cellular and humoral immune responses. The second approach was to determine whether parameters that determine kinetics of the MAP-specific immune response such as the timing and value of the peak of the immune response are predictive of the time when cows start shedding MAP. Finally, the third approach was to investigate whether a simple mathematical model (described below) is able to explain the kinetics of MAP shedding as the function of the MAP-specific cellular and humoral immune responses.

In the first approach, for calculating correlations between experimentally measured magnitude of the MAP-specific cellular lymphoproliferative T cell response (LPT), antibody response (ELISA), and MAP shedding we used Spearman Rank test. We performed two types of the analysis. In the first, conservative analysis we only calculated correlations between data for time points in which all 3 variables (LPT, ELISA, MAP) were measured. In an alternative analysis, we replaced missing values for a given parameter at a given time point by a value predicted by a linear interpolation between two adjacent measurements. This was done because shedding and immune response measurements were not always done on the same day. Results from both analyses were similar although fewer statistically significant correlations were found using the conservative (first) method. Here we reported results of the alternative analysis and to correct for type I errors we used a more stringent cut-off for statistically significant correlations (*p* = 0.002).

In the second approach, we first estimated parameters determining the kinetics of the MAP-specific cellular and humoral immune response, and of shedding. For that we used 3 different models. To describe the dynamics of the MAP-specific cellular immune response we used a so-called “*T*_on_-*T*_off_” mathematical model which was previously proposed to describe kinetics of virus-specific CD8 T cell responses in mice [27–29]. In this model immune response starts with *C*_*0*_ antigen-specific T cells immediately after infection, so *T*_on_ = 0 in this model [29]. Antigen-specific T cell response expands at a rate *ρ*_*c*_ until reaching the peak at the time *T*_off_^*C*^. After the peak, the immune response declines at a rate *δ*_*c*_. With these assumptions, the dynamics of the MAP-specific cellular (LPT) response is given by the following equation:

1 $$ \frac{dC}{dt}=\left\{\begin{array}{c}\hfill {\rho}_cC,\kern5.2em \mathrm{if}\ t<{T}_{\mathrm{off}}^C,\hfill \\ {}\hfill -{\delta}_cC,\kern5.2em \mathrm{if}\kern0.29em t\ \ge {T}_{\mathrm{off}}^C,\hfill \end{array}\right. $$

where the immune response reaches the peak *C*_max_ at time *t* = *T*_off_^*C*^. We fitted this model (Equation 1) to raw (untransformed) data (LPT). Confidence intervals for the estimated parameters were calculated using bootstrapping of the data [30]. As summarizing parameters for the LPT response, we took the level of the peak response (*C*_*max*_), the time of the peak (*T*_off_^*C*^), and the rate of decline after the peak (δ_*C*_).

To describe the dynamics of the antibody responses, we used a descriptive model based on the observed pattern in most animals: an initial constant level, followed by a linear increase, and ending in a higher constant level:

2 $$ H(t)=\left\{\begin{array}{c}\hfill {H}_0,\kern8.25em \mathrm{if}\ t<{T}_{\mathrm{on}}^H,\hfill \\ {}\hfill {H}_0+{H}_{rise}\frac{t-{T}_{\mathrm{on}}^H}{T_{\mathrm{off}}^H-{T}_{\mathrm{on}}^H},\kern3.2em \mathrm{if}\ {T}_{\mathrm{on}}^H\le t<{T}_{\mathrm{off}}^H,\hfill \\ {}\hfill {H}_0+{H}_{rise},\kern5.75em \mathrm{if}\ t\ge {T}_{\mathrm{off}}^H.\kern1em \hfill \end{array}\right. $$

This model was fitted to raw (untransformed) OD data (ELISA). As summarizing parameters for the ELISA response, we took the initial level (*H*_0_), the time at which the increase starts (*T*_on_^*H*^), and the level of increase (*H*_*rise*_).

To describe the dynamics of MAP shedding, we used a descriptive model similar to the model for the ELISA dynamics, only with a stepwise increase:

3 $$ B(t)=\left\{\begin{array}{c}\hfill {B}_0,\kern8em \mathrm{if}\ t<{T}_{\mathrm{on}}^B,\kern0.75em \hfill \\ {}\hfill {B}_0+{B}_{rise},\kern4em \mathrm{if}\ t\ge {T}_{\mathrm{on}}^B.\kern0.5em \hfill \end{array}\right. $$

This model was fitted to the (semi-quantitative) log-transformed shedding data using the following relationships: “0” - 1 bacterium, “+” - 10 bacteria, “++” – 100 bacteria, “+++” – 1000 bacteria per gram of the fecal sample. As summarizing parameters for MAP shedding, we took the initial level (*B*_0_), the time of the increase (*T*_on_^*B*^), and the level of the increase (*B*_*rise*_).

We tested associations between the nine parameters (three parameters per measured variable) by taking the following steps:

1. bootstrapping the datasets and obtaining 1000 sets of the nine parameters for all 20 animals;
2. testing the distributions of the parameters for normality by the Shapiro-Wilk test, resulting in 1000 *p*-values for each parameter. If more than 5% of *p*-values were smaller than 0.01, data were transformed to improve normality (specific transformations used are indicated in the text); if that appeared impossible, the data were dichotomized. Dichotomization was necessary for *T*_on_^*B*^ and *T*_on_^*H*^;
3. testing the associations between the parameters by Pearson’s correlation or Student’s *t*-test (with the dichotomized parameter). This resulted in 1000 association tests per couple of parameters, one for each bootstrap sample. We chose to report associations between parameters if more than 200/1000 were significant (*p* < 0.05). In that case, we report the direction of the association and the number of significant associations in the 1000 bootstrap samples to indicate the certitude about this association.

In the third approach, to investigate how and whether immune response as measured by LPT and ELISA contributed to the kinetics of bacteria shedding in feces we used a simple mathematical model. In the model we assumed that shedding *B*(*t*) changes over time at a per capita rate *r* and that both cellular (*C*, LPT) and humoral (*H*, ELISA) immune responses influence change in shedding over time at per capita rates *k*_*C*_ and *k*_*H*_, respectively. With these assumptions, the change in bacterial shedding over time is given by the model

4 $$ \frac{dB(t)}{dt}=rB(t)-\left({k}_CC(t)+{k}_HH(t)\right)B(t). $$

To describe the kinetics of MAP-specific immune responses, *C(t)* and *H(t)*, we used interpolation function *Interpolation* in Mathematica 5.2 with interpolation order 1. More specifically, we connected measured values for the immune responses using linear interpolation for Log10 of LPT data (*C*) or linear interpolation of ELISA data (*H*) which effectively allowed us to have predicted measurements for the immune responses at any point of time within our measurements. We also tested if shedding can be predicted equally well by using the *T*_on_-*T*_off_ model for the LPT response (Equation 1) using Akaike Information Criterion (AIC) and Akaike weights as described in [31]. Shedding data were transformed from categorical data to bacterial counts as described above. Model predictions given in Equation 4 were log10 transformed and compared to the experimentally measured bacterial counts. In the model we assumed that maximal density that can be measured in the animals is *B*_*max*_ = 10^3^ cfu/g so model solutions resulting in higher densities were set to be equal to *B*_*max*_. We used nonlinear least squares to find the best fit parameters *B*_*0*_ (initial shedding), *r*, *k*_*C*_, and *k*_*H*_. Four different subsets of the main model (Equation 4) were fitted to data: only growth (*B*_0_ and *r*), Th1 control (*B*_0_, *r*, *k*_C_), Th2 control (*B*_0_, *r*, *k*_H_), and Th1/Th2 control (*B*_0_, *r*, *k*_C_, *k*_H_). We determined the best fit model using the F-test for nested models [32] and accepted the more complex model if there was a statistically significant improvement of model fit to data. If all models resulted in similar quality fits, the simplest model (with fewest parameters) was preferred, and in the case of models with the same number of parameters, the model with the highest Akaike weight was chosen [31].

## Results

### Experimental details

All animals became infected as judged by frequent presence of the bacteria in feces but kinetics of the shedding over time varied between animals. All animals showed a biphasic LPT response characterized by an initial exponential increase, followed by an exponential decrease, and many animals showed an ELISA response, characterized by an increase in OD value after a variable delay (Additional file 5).

### Correlations between immune response and shedding

**Figure 2:**
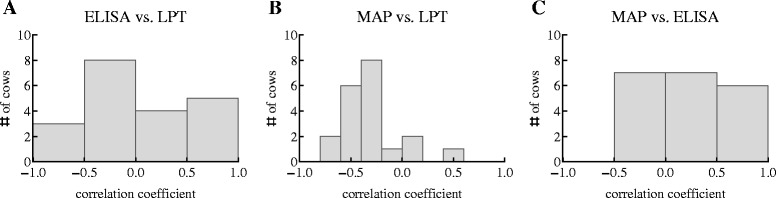
**Highly variable relationships between shedding (MAP) and MAP-specific cellular (LPT) and humoral (ELISA) immune responses.** We calculate correlation coefficients (using Spearman rank test) between measurements of humoral and cellular immunity (panel A), between shedding and cellular immunity (panel B), and shedding and humoral immunity (panel C). Because not all data were available at all time points, missing values were generated using linear interpolations (see Materials and methods for detail). Numerical values for the estimated correlation coefficients are given in Additional file 1. The histograms of the correlation coefficients were similar with fewer correlations being statistically significant if we restrict our analysis to data where all 3 variables (LPT, ELISA, MAP) were measured at the same time (results not shown).

To investigate relationships between measured responses and shedding, we calculated Spearman rank correlations between magnitudes of the immune responses (ELISA vs. LPT), between shedding and cellular immunity (MAP vs. LPT), and between shedding and humoral immunity (MAP vs. ELISA, see Figure 2 and Additional file 1). We found both negative and positive correlations between these variables in our 20 animals (Figure 2). In particular, we found both positively and negatively correlated MAP-specific cellular and humoral immunity challenging the common view of “competition” between these responses in MAP infection (e.g. [21]). This conclusion remained valid if we restricted our analysis to statistically significant correlations (at the level of *p* = 0.002 to correct for multiple comparisons). Of note, only 6 correlations between LPT and ELISA levels out of 20 animals were statistically significant (3 correlations were positive and 3 were negative, see Additional file 1) suggesting that in most animals, there was no evidence of competition or synergy between MAP-specific cellular and humoral immunity in contrast with the prevailing dogma on exclusiveness of these types of responses.

The situation was somewhat different when we looked at correlations between shedding and immune responses. Here, cellular immunity (LPT) was strongly negatively correlated with shedding while humoral immunity was positively correlated with shedding. This was in line with expectations that cellular immune response may be involved in the control of immunity while humoral immunity may either indicate the increase in shedding [21] or may be assisting with increase in shedding, e.g., by enhancing infection of macrophages by MAP [18]. However, many correlations were not statistically significant (Additional file 1).

### Kinetics of the MAP-specific immune responses and shedding

In the next step, we summarized the LPT, ELISA, and MAP dynamics by three parameters per variable, and tested for associations between these parameters across cows.

**Figure 3:**
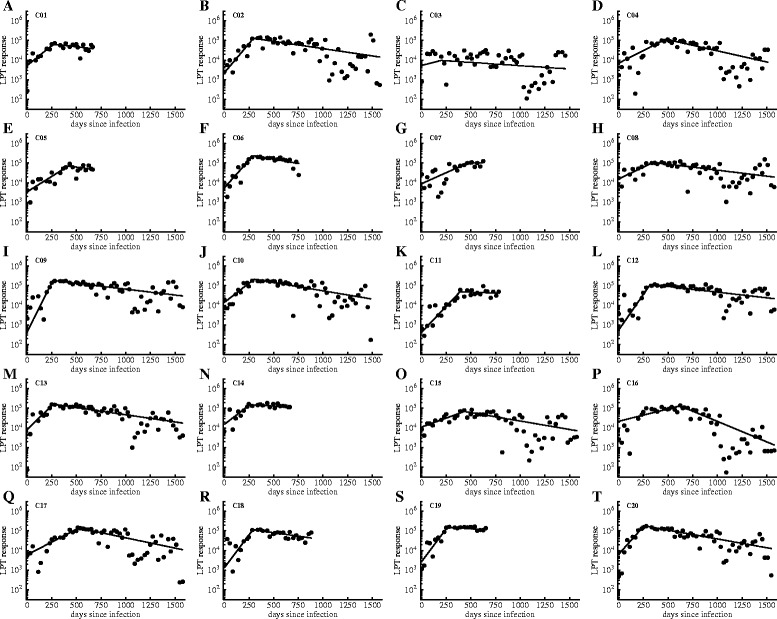
**Kinetics of the MAP-specific cellular immune response and fits of the mathematical model to these experimental data.** We measured MAP-specific cellular immune response using lymphoproliferative assay detecting IFNγ-producing, MAP-specific T cells following infection of calves with MAP (LPT data, Materials and methods for more detail). We fitted a simple, “T_on_-T_off_” model in which immune response expands to a peak and then contracts (see Equation 1) to the raw LPT data. Results were similar if we fitted log-transformed model predictions to the log-transformed data although some parameter estimates changed (results not shown). Labels for individual cows are shown in the top left corner. Data are shown as points and model predictions are given by lines. Parameters of the best fit model to these data are shown in Additional file 2.

**Figure 4:**
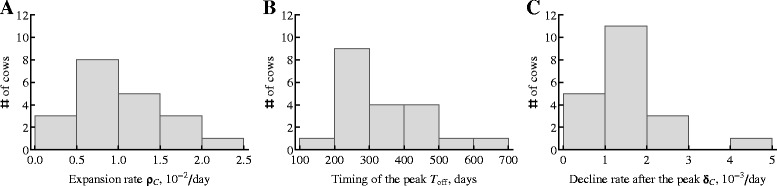
**Distribution of estimated parameters characterizing kinetics of the MAP-specific cellular immune response.** We fitted a simple “*T*_on_-*T*_off_” model to the experimental LPT data (fits are shown in Figure 3) and plotted the distribution of estimated parameters. Shown parameters are the rate of expansion of the immune response over time ρ_*C*_ (panel **A**), the time of the predicted peak of the immune response *T*_off_^*C*^ (panel **B**), and the rate of decline of the immune response after the peak δ_*C*_ (panel **C**; see Additional file 2 for the list of parameter estimates and their 95% confidence intervals).

To quantify the kinetics of the T cell response to MAP in cattle we fitted a simple mathematical model to the LPT data (Figure 3). As was expected from the clonal selection theory, MAP infection led to an exponential increase in the size of the MAP-specific cellular immune response; this increase continued for several months (Figure 3). The median expansion rate ρ_C_ was 0.009 per day and the response peaked on average 1 year post infection (Figures 3 and 4). After reaching the peak, there was a consistent loss of the MAP-specific cellular immunity in most animals but the actual rate of loss was highly variable between animals. The median loss rate was 0.0015 per day implying 1.3 year half-life time for MAP-specific T cell response. For the correlation analyses, we used the level of the peak (*C*_*max*_), the time of the peak (*T*_off_^*C*^), and the rate of decrease (δ_*C*_), estimated for each cow (+/− 95% bootstrap percentiles) (Additional file 3). For the association analyses, the parameters needed to be normally distributed across cows, which was tested by the Shapiro-Wilk test on each bootstrap sample. Normality was rejected (*p* < 0.01) in 0/1000 bootstrap samples for *C*_*max*_, in 198/1000 samples for *T*_off_^*C*^, and in 414/1000 samples for δ_*C*_. Because exponential transformation of δ_*C*_ reduced the rejected rate to 20/1000, we applied this transformation for the association analysis. No improvement was possible for *T*_off_^*C*^ by transformations, so we dichotomized these data into cows with *T*_off_^*C*^ < 300 or *T*_off_^*C*^ ≥ 300 to obtain approximately equally-sized groups.

We fitted a descriptive model to the ELISA data. The model given in Equation 2 consisted of three linear segments including the initial level (*H*_0_), the time of increase of response (*T*_on_^*H*^), and the level of increase (*H*_*rise*_). This model can be treated as a “*T*_on_-*T*_off_” model under the assumption that OD values measured by ELISA represent log-transformed titers of MAP-specific antibodies and that there is no loss of MAP-specific humoral immunity after the peak. The model described the kinetics of the response in all animals well (see Additional file 6). Bootstrap medians and 95% percentiles are given in Additional file 3. Normality was rejected (*P* < 0.01) in 3.7%, 7.9%, and 88.5% of bootstrap samples for *H*_0_, *T*_on_^*H*^, and *H*_*rise*_, respectively, so *H*_0_ was not transformed. No improvement was possible for *T*_on_^*H*^ by transformations, so we dichotomized these data into cows with *T*_on_^*H*^ < 400 or *T*_on_^*H*^ ≥ 400 to obtain approximately equally-sized groups. Log10-transformation of *H*_*rise*_ was used for normalization, reducing the rejection rate to 13/1000.

**Figure 5:**
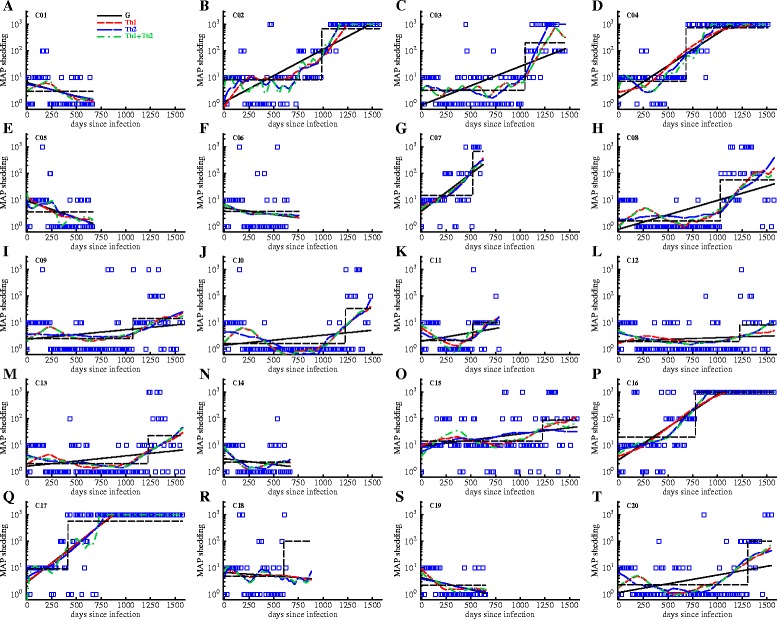
**MAP-specific cellular and humoral immune responses improve description of MAP shedding in most animals.** We fitted four subsets of the mathematical model (Equation 4) to the experimentally measured shedding levels (shown by markers) and estimated model parameters. Predictions of the models are shown by lines. The four models were G: exponential growth in shedding (solid black line), Th1: exponential growth in shedding with suppression by Th1 (LPT) response (dashed red line), Th2: exponential growth in shedding with suppression by Th2 (ELISA) response (dashed blue line), Th1 + Th2: exponential growth in shedding with suppression by both Th1 (LPT) and Th2 (ELISA) responses (dashed-dotted green line). The parameters of the best fit model are shown in Table 2 and Akaike weights of different models are shown in Additional file 4. We also show fits of the simple “switch” model (Equation 3) to the shedding data by thin dashed lines. Parameters for this model are given in Additional file 3.

We fitted another descriptive model to the MAP shedding data. The model included the initial MAP level (*B*_0_), the time of increase of shedding (*T*_on_^*B*^), and the level of increase (*B*_*rise*_) (Figure 5). Bootstrap medians and 95% percentiles are given in Additional file 3. Normality was rejected (*p* < 0.01) in 179/1000, 19/1000, and 19/1000 bootstrap samples for *B*_*0*_, *T*_on_^*B*^, and *B*_*rise*_, respectively, so *T*_on_^*B*^ and *B*_*rise*_ were not transformed. Log10-transformation of *B*_*0*_ (adding 0.01 to prevent log10(0)) reduced the rejection rate to 25/1000.

### Impact of the immune response parameters on the kinetics of shedding

Our analysis revealed that only a small subset of parameters characterizing kinetics of the MAP-specific immune responses and MAP shedding were correlated at a statistically significant level (Table 1). For all three variables (LPT, ELISA, and MAP), one parameter was not associated to any other parameter, whereas the other two were mutually associated: *T*_off_^*C*^ and δ_*C*_; *H*_0_ and *H*_*rise*_; *B*_0_ and *T*_on_^*B*^. These associations can be illustrated by considering two extreme scenarios of disease development in MAP-infected cows.

**Table 1:** Associations between parameters summarizing the course of the cellular and humoral immune responses and MAP shedding

	*C*_max_	*T*_off_^*C*^	δ_C_	*H*_0_	*T*_on_^*H*^	*H*_*rise*_	*B*_0_	*T*_on_^*B*^	*B*_*rise*_
*C*_max_ (cpm)		'-' 21.5%							
*T*_off_^*C*^ (days)				'+' 75.3%		'+' 27.9%		'-' 29.7%	
δ_C_ (per day)						'+' 33.7%			
*H*_0_ (% OD)						'+' 73.9%		'-' 31.8%	
*T*_on_^*H*^ (days)									
*H*_*rise*_ (% OD)							'+' 76.6%	'-' 91.7%	
*B*_0_ (log(cfu/g))								'-' 23.4%	
*T*_on_^*B*^ (days)									

The parameters used in the analysis: *C*_max_ is the peak value for the cellular immune response, *T*_off_^*C*^ is the time of the peak, δ_C_ is the rate of decrease of the cellular immune response after the peak, *H*_0_ is the initial value of the antibody response, *T*_on_^*H*^ is the time of start of increase in OD value, *H*_*rise*_ is the amount of increase in OD value, *B*_0_ is the initial shedding, *T*_on_^*B*^ is the predicted time of the increase in shedding, and *B*_*rise*_ is the amount of increase in MAP shedding. Positive ('+') and negative ('-') associations are indicated if at least 20% bootstrapped datasets showed a significant association (*p* < 0.05); the exact percentage is given. Parameter Brise was measured in log(cfu/g) units.

The one extreme is characterized by low initial shedding and a late increase to high shedding; this is associated with an early LPT peak, a slow decline in LPT response after the peak, and a low ELISA response. The other extreme is characterized by high initial shedding and an early increase to high shedding; this is associated with a late LPT peak, a rapid decline in LPT response after the peak, and a high ELISA response. It should be noted, however, that while we observed the correlations between different parameters for the MAP and immune response dynamics, they appeared to be significant only in a subset of bootstrap samples. Only the negative correlation between *H*_*rise*_ and *T*_on_^*B*^ appeared statistically significant in most examined bootstraps (Table 1).

### Using mathematical modeling to predict impact of immunity on shedding kinetics

Our analyses so far demonstrated that there was a correlation between MAP-specific cellular immunity and timing of the shedding in MAP-infected cattle (Table 1). In particular, a longer expansion phase of the MAP-specific T cell response (*T*_off_^*C*^) was inversely correlated with the time of high shedding (*T*_on_^*B*^) implying that longer duration of the Th1 response led to an earlier rise in bacterial shedding. However, it was still unclear if immunity could explain the overall pattern of change in shedding over time. All of the exposed animals displayed some degree of shedding but some animals were able to control shedding at a low level (e.g., C12) while others rapidly progressed to high shedding (e.g., C07). To get further insights into factors controlling overall kinetics of shedding we developed a simple mathematical model and fitted that model to the experimental data (see Equation 4 and Materials and methods for more details). In the model we assumed that in the absence of the immune response bacterial shedding increases exponentially over time although we did allow for spontaneous control of shedding by the adaptive immunity-independent mechanisms (i.e., we allowed the rate of increase in shedding over time *r* to be negative). The simple, “exponentially growing shedding” model could explain reasonably well change in shedding over time in several animals: mainly those that died early due to MAP-unrelated reasons (and thus these animals appeared to control MAP replication efficiently, e.g., C01) or progressed rapidly to the stage of high shedding (e.g., C07; see Figure 5 and Table 2). In most animals, however, inclusion of the immune response resulted in statistically improved fits of the model to the shedding data (Table 2 and Additional file 4). Cellular immunity was required to explain shedding data in 8 out of 20 animals while humoral immunity was required to explain shedding data in 9 out of 20 animals (Table 2). Interestingly, we found that contribution of immunity seemed not always protective as many estimated “killing” constants were negative (Table 2). In fact, only in half of animals in which cellular immune response as measured by LPT was required for best description of the shedding data, cellular immunity was protective (estimate *k*_C_ > 0, Table 2). In the remaining animals, cellular immunity was predicted to increase shedding (e.g., C02, C11). In contrast, humoral immunity was predicted to be nearly always detrimental: in all but one animal, the model predicted that an increase in antibody titers resulted in higher shedding (e.g., C02, C04; Table 2). Notably, in many animals we predicted a negative growth in shedding (r < 0) suggesting that other mechanisms besides those indicated by blood LPT and ELISA responses may be influencing early control of MAP replication. These results suggest that both cellular or humoral immunity could be promoting the disease/shedding of MAP in infected animals, rather than containing and clearing the infection.

**Table 2:** Estimates of parameters providing the best fit of the mathematical model to the shedding data

cow ID	B_0_, CFU/g	*r*, 10^−2^/day	*k*_C_, 10^−7^/day	*k*_H_, 10^−3^/day
C01	6.3	−0.2	0	0
C02	4.7	0.8	−2.55	−571.49
C03	3.6	−0.4	0	−76.32
C04	4.2	0.1	2.17	−189.83
C05	9.2	−0.3	0	0
C06	5	−0.1	0	0
C07	3.9	0.6	0	0
C08	1	1.2	1.69	0
C09	2.2	0.6	0.64	0
C10	4.2	0	0	−76.71
C11	8.3	0	−6.9	76.24
C12	4.1	−0.2	0	−11.27
C13	4.2	−0.2	0	−16.06
C14	8.7	−1.3	−0.99	0
C15	9.7	0.4	1.03	0
C16	6.6	0.1	0	−9.15
C17	0.6	0	4.69	−165.86
C18	6.7	−0.1	0	0
C19	9.1	−0.9	−0.63	0
C20	6.4	−0.4	0	−64.95

The best fit model (the model is shown in Equation 4) was determined by performing F-tests for nested models and using Akaike weights for non-nested models (see Materials and methods for more detail and Additional file 4). Listed parameters are: *B*_0_ is the predicted initial shedding level, *r* is the rate of exponential change in shedding in the absence of MAP-specific cellular (as measured by LPT) and humoral (as measured by ELISA) immune responses, *k*_C_ is the predicted rate of reduction in shedding by the cellular immune response, *k*_H_ is the predicted rate of reduction in shedding by the humoral immune response. Negative values in shedding rate change *r* indicate predicted decline in shedding in the absence of measurable cellular and antibody responses. Negative values in *k*_C_ and *k*_H_ indicate increase in shedding with the presence of corresponding immune responses.

## Discussion

Experimental infection of calves with MAP led to variable disease outcomes. Some animals were able to control bacteria and display low degree of shedding (e.g., C09, C12) while other animals progressed to the state of high shedding (e.g., C02, C04) in the 5 year observation period. It remains unclear, however, whether animals that displayed low to average levels of shedding would have remained low shedders or whether their immune system would have failed allowing uncontrolled bacterial growth and high shedding. Factors influencing the rate at which infected animals progress to clinical disease and become high shedders are currently incompletely understood. Our analysis provides some novel insights into such factors.

Since MAP is a mycobacterium and is able to infect and replicate in macrophages [3], there is an expectation that cellular, mainly CD4 T cell response, should be contributing to the containment of the bacteria and delaying disease progression [33,34]. Because a MAP-specific antibody response usually develops late in the infection when cellular immunity declines, it has been suggested that there is an active competition between cellular and humoral immunity, the so-called Th1-Th2 switch [13,20,21]. Therefore, over the course of disease progression there is an expectation that MAP shedding should be inversely related to the magnitude of the MAP-specific cellular immune response and there should be negative correlation between cellular (Th1) and humoral (Th2) immunity [21]. Our correlation-based analysis confirmed and rejected some of these ideas. Indeed, most of the correlations between shedding and cellular immunity were negative and between shedding and humoral immunity were positive. Yet, many of these correlations were not statistically significant even in the well-sampled longitudinal study as ours (Figure 2). Furthermore, the correlation between magnitude of cellular and humoral immunity was negative only half the time suggesting that the previously argued competition between Th1 and Th2 responses in MAP infection may be an artifact of the analysis of small datasets. Indeed, recently it was found that the loss of MAP-specific cellular immunity and rise in MAP-specific antibodies was observed in only 40% of MAP-infected sheep, with other sheep either displaying both responses simultaneously or only cellular immunity [35]. It should be emphasized that observing statistically significant correlations does not imply causality, and in fact, the absence of significant correlations in most animals may indicate that these associations emerge in other animals due to confounding, e.g. with the age of the animal.

Some of the shortcomings of the correlation analysis were corrected by looking only at long-term average values such as decay rates and times at which these rates change (Table 2). There was a great variability in shedding within individual animals over time where sometimes high and low MAP densities were found on 2 consecutive measurements (Additional file 1). Furthermore, the rate of increase over time was also highly variable between animals. Due to this variability, very few correlations turned out to be significant. Because all infections were initiated by the same dose, this observed variability may reflect the stochastic nature of disease progression in animals. Variable outcomes of disease progression were also found during MAP infection of sheep [35]. We found a strong correlation between the increase in MAP-specific antibodies and the time of high shedding suggesting that humoral immunity is unlikely to play a strong role in controlling bacterial shedding. This result in fact suggested that high antibody responses may be the consequence of the high level of shedding, and not the actual cause of the increased shedding. Interestingly, in a subset of animals, a later peak of the MAP-specific cellular immunity *T*_off_^*C*^ was associated with an earlier switch to high shedding *T*_on_^*B*^ (Table 1). If this later peak indicates a longer response, it could indicate that cellular immunity may promote, rather than contain high shedding, but if the later peak indicates later initiation of the response, it would confirm the active role of cellular immunity in containing MAP. The major problem with this second approach, however, was the lack of power, because this analysis basically reduces the number of independent observations to 20, which did not permit strong statistically founded conclusions. Indeed, most entries in Table 1 are empty, i.e. not statistically significant.

The correlation analyses (approaches 1 and 2) still did not allow investigation of the cause-and-effect relationship between various parameters. To partially address this problem we used a simple mathematical model which predicted change in shedding over time as the function of MAP-specific immune responses. The overall message of this analysis was that immune responses significantly contributed to the change in shedding over time in most animals; yet, the direction of influence was different between animals, and sometimes depended on the constraints put on the model structure and model parameters. For example, by allowing shedding to be spontaneously controlled (allowing *r* < 0), we found that in many animals a stronger immune response increased shedding (*k*_C_ < 0 and *k*_H_ < 0). This was mainly observed in animals which controlled MAP shedding early in infection but died due to MAP-unrelated reasons (e.g., C01, C05). Alternatively, fitting a model in which shedding cannot decrease on its own (allowing only *r* > 0) forced immunity to have controlling effect on shedding in such animals (*k*_C_ > 0, results not shown). Importantly, however, even when shedding cannot decrease in the absence of immunity, the model fits predicted that MAP-specific cellular immune response still contributed positively to shedding in several animals. This analysis raised an interesting possibility that MAP-specific T cell responses by producing INFγ may not only restrain bacterial replication, but may also contribute to the pathology and could in fact increase severity of shedding. Recent work on *Mycobacterium tuberculosis* infection of mice also suggested that immune responses may be drivers of pathogenesis [36,37]. In contrast, MAP-specific antibodies as measured by ELISA nearly always contributed to the increase in shedding (*k*_C_ < 0, Table 2). The positive association between MAP-specific antibodies and shedding is a consistent result in all of our analyses and was also recently observed in animals infected naturally on farms [38]. Our mathematical model-based fits of the shedding data suggest that MAP-specific antibodies enhance shedding, but it should be noted that an alternative model in which shedding increases antibody response e.g., [20] was not analyzed and may also be an explanation. The relationship between shedding and antibody response could be investigated using antibiotic treatment of animals with high antibody titers. Reduction in shedding following treatment should not impact the levels of MAP-specific antibodies if antibodies drive bacterial shedding.

We used several nested mathematical models to investigate the contribution of MAP-specific immune responses to bacteria shedding. It is clear, however, that the most general model (Equation 4 with *k*_*C*_ ≠ 0 and *k*_*H*_ ≠ 0) was still extremely simple and did not capture the biological complexity of the interactions between immunity and bacteria within individual animals. An alternative approach could be to use a more mechanistic model that incorporates explicitly dynamics of bacteria and MAP-specific Th1 and Th2 responses [20]. We found, however, that it was difficult to adequately fit such a model to all 3 types of data (LPT, ELISA, MAP) without making additional assumptions regarding mechanisms of interactions between responses and bacteria (results not shown). It is nevertheless possible that some of our results stemmed from the type of the model we used; for example, we did find different predictions for the model parameters *k*_C_ and *k*_H_ if the growth rate of shedding was constrained to be positive. Another potential problem was our assumption that the difference between model predictions and shedding data followed a normal distribution, whereas shedding data were only given in four categories. It is possible that more complex methods such as time-dependent hazard survival analysis would have been more appropriate, but it is unlikely that it would have affected the variability in associations across the animals.

Although it was not the primary goal, our analysis also provided basic estimates of parameters characterizing kinetics of cellular and humoral immune responses to intracellular pathogens such as MAP. We found that the median rate of exponential expansion of MAP-specific T cell response was 0.009/day implying a 75 days doubling time, resulting in on average only 5 divisions until the peak. This is an extremely slow and limited expansion compared to estimates for various viral infections in mice, humans, and monkeys, with doubling times ranging from 6 h to a couple of days [27–29,36,38,39]. A slow rate of expansion of virus-specific T cell responses during chronic infections has been noted and discussed previously [39]. Because MAP-infected cows were housed together and were likely to get re-exposed to infection regularly, it remains to be investigated whether such constant re-exposure was the reason for the slow kinetics of the MAP-specific cellular immune response. Alternatively, the slow expansion of T cell populations in blood could be due to sequestration of MAP-specific T cells in tissues; additional longitudinal data on the T cell dynamics in tissues are needed to test this hypothesis.

In conclusion, we presented results of a unique experiment following MAP infected cows for up to 5 years, with frequent measurements of shedding and cellular and humoral immune responses. The lack of strong correlations that were the same in all or most cows illustrated that the role of the immune responses in controlling the infection was limited, or even absent. Cellular immunity may exert some control over the infection in some animals (but seems to stimulate in others), and humoral immunity generally seemed to be positively related to MAP shedding, or not at all. If the adaptive immune response had indeed a relatively limited a role in dynamics of the infection, an important question for future research will be to find the mechanisms that do cause the long subclinical period and large differences in infection outcome between cows.

## Additional files

Additional file 1:
**Observed correlations between MAP-specific cellular immune response (measured by LPT), MAP-specific humoral immune response (measured by ELISA), and MAP shedding (MAP).** We list correlation coefficients calculated using nonparametric Spearman rank correlation test. Correlation coefficients with *p*-values lower than 0.002 (to correct for multiple comparisons) are highlighted with a star “*”. The number of statistically significant positive and negative correlations is shown in the last two rows. Additional file 2:
**Parameters of the “T**_**on**_ **-T**_**off**_ **” model fitted to the LPT data.** We fitted the simple mathematical model (Equation 1 in the main text), which describes expansion and contraction of cellular immune response, to the LPT data using nonlinear least squares and estimated model parameters. Ninety five (95%) confidence intervals (shown in bracket) were obtained by bootstrapping the data and refitting the model 1000 times in each animal. Additional file 3:
**Parameters (medians and 95% percentiles) used for the association analyses.** If no result is given, no ELISA or MAP increase was observed and/or the final ELISA or MAP level was not reached during the experiment. Additional file 4:
**Akaike weights of the 4 different subsets of models fitted to the MAP shedding data.** Models are “G” (fitting only *B*_0_ and *r*), “Th1” (fitting *B*_0_, *r*, *k*_C_), “Th2” (fitting *B*_0_, *r*, *k*_H_), and “Th1 + Th2” (fitting *B*_0_, *r*, *k*_C_, *k*_H_). The model is given in Equation 4 in the main text and details on the fitting and the definition of the best model are described in Materials and methods. Best model is highlighted in bold. Additional file 5:
**Experimental data for 20 calves infected with MAP.** Circles denote MAP-specific cellular immunity (as measured by LPT), triangles denote MAP-specific humoral immunity (as measured by ELISA), and boxes are for MAP shedding in faeces. The Y-axis label “density” represents cpm for LPT response, OD405nm values for ELISA (with 10^x^ shown where x is the OD recorded in the assay, see Additional file 6), and shedding level for MAP (with categorical data being transformed as described in Materials and methods in the main text). Additional file 6:
**Levels of MAP-specific antibodies and fits of the mathematical model (Equation** **3** **in the main text) to these data.** ELISA response is shown as PPD-P specific background corrected optical densities (OD405nm) on the Y-axis. Parameters of the model are given in Additional file 3.
