# Supplementary material for: Evaluating contribution of the cellular and humoral immune responses to the control of shedding of Mycobacterium avium spp. paratuberculosis in cattle
Source: Vet Res. 2015 Jun 19;46(1):62. doi: 10.1186/s13567-015-0204-1 (PMC4474352; doi:10.1186/s13567-015-0204-1)
Supplement: Additional file 1: — Observed correlations between MAP-specific cellular immune response (measured by LPT), MAP-specific humoral immune response (measured by ELISA), and MAP shedding (MAP). We list correlation coefficients calculated using nonparametric Spearman rank correlation test. Correlation coefficients with p-values lower than 0.002 (to correct for multiple comparisons) are highlighted with a star “*”. The number of statistically significant positive and negative correlations is shown in the last two rows. [file 13567_2015_204_MOESM1_ESM.docx]

| cow ID | LPT vs. ELISA | LPT vs. MAP | ELISA vs. MAP |
| --- | --- | --- | --- |
| C01 | 0.455 | -0.446* | -0.428 |
| C02 | -0.51* | -0.506* | 0.589* |
| C03 | -0.42 | -0.286 | 0.526* |
| C04 | -0.294 | -0.423* | 0.639* |
| C05 | 0.14 | -0.35 | -0.424 |
| C06 | 0.789* | -0.393 | -0.292 |
| C07 | 0.519 | 0.438 | 0.075 |
| C08 | -0.59* | -0.616* | 0.607* |
| C09 | -0.348 | -0.609* | 0.288 |
| C10 | -0.566* | -0.428* | 0.251 |
| C11 | 0.65* | 0.011 | 0.305 |
| C12 | -0.136 | -0.215 | 0.024 |
| C13 | -0.397 | -0.332* | 0.112 |
| C14 | 0.65* | -0.363 | -0.173 |
| C15 | 0.143 | -0.153 | 0.147 |
| C16 | -0.391 | -0.46* | 0.762* |
| C17 | -0.185 | 0.077 | 0.744* |
| C18 | 0.269 | -0.287 | -0.022 |
| C19 | 0.102 | -0.514* | -0.162 |
| C20 | -0.187 | -0.33* | -0.03 |
| # of positive correlations | 3 | 0 | 6 |
| # of negative correlations | 3 | 10 | 0 |
