# Supplementary material for: Evaluating contribution of the cellular and humoral immune responses to the control of shedding of Mycobacterium avium spp. paratuberculosis in cattle
Source: Vet Res. 2015 Jun 19;46(1):62. doi: 10.1186/s13567-015-0204-1 (PMC4474352; doi:10.1186/s13567-015-0204-1)
Supplement: Additional file 2: — Parameters of the “T on -T off ” model fitted to the LPT data. We fitted the simple mathematical model (Equation 1 in the main text), which describes expansion and contraction of cellular immune response, to the LPT data using nonlinear least squares and estimated model parameters. Ninety five (95%) confidence intervals (shown in bracket) were obtained by bootstrapping the data and refitting the model 1000 times in each animal. [file 13567_2015_204_MOESM2_ESM.docx]

| Cow ID | *C*_0_, 10^3^ cpm | *ρ_C_*, 10^-3^/day | $T_{\text{off}}^{C}$, day | *δ*_C_, 10^-3^/day |
| --- | --- | --- | --- | --- |
| C01 | 5.33 (1.86-12.00) | 9.34 (4.37-14.68) | 267 (233-391) | 1.287 (0.078-3.376) |
| C02 | 2.29 (4.44 10^-7^-14.57) | 13.67 (4.51-75.33) | 295 (254-469) | 1.713 (0.702-2.654) |
| C03 | 5.37 (0.78-51.72) | 2.97 (-6.49-329.29) | 198 (10-665) | 0.764 (0.000-3.599) |
| C04 | 7.11 (0.00027-17.44) | 5.88 (3.61-45.02) | 475 (290-555) | 2.597 (1.856-3.754) |
| C05 | 3.45 (0.23-8.54) | 7.24 (3.39-14.39) | 416 (364-624) | 1.410 (0.000-12.330) |
| C06 | 5.51 (0.22-13.77) | 12.93 (8.00-27.07) | 291 (260-378) | 1.962 (1.026-3.190) |
| C07 | 8.92 (0.000055-20.88) | 5.18 (3.24-50.69) | 470 (280-550) | 0.000 (0.000-14.540) |
| C08 | 15.64 (2.64-28.67) | 5.56 (3.23-12.65) | 349 (281-451) | 1.405 (0.601-2.331) |
| C09 | 0.45 (0.00014-7.93) | 23.04 (10.06-57.47) | 261 (244-336) | 1.375 (0.867-1.870) |
| C10 | 14.11 (2.27-32.92) | 9.00 (4.42-20.11) | 302 (226-411) | 1.947 (1.483-2.676) |
| C11 | 0.43 (0.0012-2.95) | 12.13 (5.31-27.38) | 391 (391-623) | 0.460 (0.000-6.570) |
| C12 | 0.56 (0.00084-7.69) | 16.71 (7.22-42.29) | 319 (275-413) | 1.326 (0.977-1.808) |
| C13 | 8.36 (0.53-34.77) | 11.18 (3.32-23.08) | 265 (248-471) | 1.686 (1.351-2.373) |
| C14 | 15.14 (2.40-51.41) | 8.90 (2.99-16.92) | 258 (242-434) | 0.637 (0.072-2.289) |
| C15 | 11.02 (4.80-19.44) | 4.23 (1.72-7.95) | 434 (317-698) | 1.981 (1.232-3.710) |
| C16 | 21.81 (1.51-40.84) | 2.91 (1.64-14.93) | 621 (304-695) | 4.799 (2.317-7.057) |
| C17 | 6.04 (1.85-9.87) | 6.01 (4.83-9.38) | 523 (460-554) | 2.410 (1.776-3.302) |
| C18 | 1.31 (0.20-11.98) | 15.45 (5.84-22.39) | 290 (281-372) | 1.663 (0.805-2.595) |
| C19 | 2.71 (0.082-10.65) | 15.44 (7.46-30.19) | 261 (250-387) | 0.729 (0.058-1.917) |
| C20 | 6.55 (0.77-12.31) | 12.95 (10.18-24.96) | 256 (219-267) | 2.061 (1.603-2.495) |
