# Supplementary material for: Evaluating contribution of the cellular and humoral immune responses to the control of shedding of Mycobacterium avium spp. paratuberculosis in cattle
Source: Vet Res. 2015 Jun 19;46(1):62. doi: 10.1186/s13567-015-0204-1 (PMC4474352; doi:10.1186/s13567-015-0204-1)
Supplement: Additional file 3: — Parameters (medians and 95% percentiles) used for the association analyses. If no result is given, no ELISA or MAP increase was observed and/or the final ELISA or MAP level was not reached during the experiment. [file 13567_2015_204_MOESM3_ESM.docx]

|  |  | LPT |  |  | ELISA |  |  | MAP |  |
| --- | --- | --- | --- | --- | --- | --- | --- | --- | --- |
| Cow ID | *C_max_,* 10^3^ | $T_{\text{off}}^{C}$, days | *δ_C_,* 10^-3^/day | *H*_0_ | $T_{\text{on}}^{H}$*, days* | *H_rise_* | *B*_0_, log10 cfu/g | $T_{\text{on}}^{B}$*, days* | *B_rise_* |
| 1 | 66 | 265 | 0.547 | -0.038 |  |  | 0.48 |  |  |
|  | 52;82 | 236;317 | 0.08;1.207 | -0.053;-0.023 |  |  | 0.28;0.67 |  |  |
| 2 | 137 | 294 | 0.731 | -0.041 | 539 |  | 0.91 | 987 | 1.92 |
|  | 99;177 | 246;364 | 0.345;1.07 | -0.058;-0.025 | 390;845 |  | 0.71;1.12 | 889;1071 | 1.70;2.13 |
| 3 | 20 | 36 | 0.277 | 0.042 | 676 | 0.259 | 0.53 | 1050 | 1.81 |
|  | 14;26 | 4;86 | 0.029;0.502 | 0.022;0.062 | 572;938 | 0.212;0.332 | 0.37;0.70 | 1029;1151 | 1.51;2.09 |
| 4 | 116 | 477 | 1.15 | 0.086 | 936 | 0.845 | 0.85 | 683 | 2.02 |
|  | 102;142 | 400;501 | 0.856;1.58 | 0.059;0.111 | 910;1001 | 0.771;0.924 | 0.71;0.98 | 662;771 | 1.85;2.19 |
| 5 | 64 | 409 | 0.39 | 0.160 |  |  | 0.56 |  |  |
|  | 46;87 | 400;541 | 0;1.96 | 0.131;0.197 |  |  | 0.38;0.78 |  |  |
| 6 | 239 | 291 | 0.456 | 0.064 | 154 | 0.187 | 0.55 |  |  |
|  | 210;281 | 264;317 | 0.851;1.28 | 0.020;0.108 | 102;260 | 0.139;0.239 | 0.31;0.79 | ;532 | ;0.88 |
| 7 | 103 | 472 | 0.035 | 0.056 | 85 |  | 1.09 | 518 | 1.59 |
|  | 85;130 | 363;548 | 0;6.31 | 0.021;0.089 | 33;231 |  | 0.81;1.32 | 230;532 | 0.84;1.97 |
| 8 | 109 | 348 | 0.612 | -0.026 | 984 | 0.227 | 0.21 | 1029 | 1.57 |
|  | 85;130 | 281;434 | 0.234;0.972 | -0.040;-0.012 | 953;1018 | 0.185;0.273 | 0.11;0.31 | 1015;1112 | 1.25;1.88 |
| 9 | 183 | 261 | 0.596 | -0.019 | 923 | 0.178 | 0.39 | 1071 | 0.77 |
|  | 152;210 | 219;330 | 0.363;0.832 | -0.031;-0.008 | 828;953 | 0.148;0.218 | 0.24;0.56 | 802;1219 | 0.45;1.11 |
| 10 | 215 | 302 | 0.843 | -0.029 | 933 | 0.147 | 0.22 | 1226 | 1.41 |
|  | 191;250 | 243;371 | 0.650;1.11 | -0.040;-0.012 | 905;947 | 0.115;0.183 | 0.12;0.34 | 1194;1324 | 0.89;2.05 |
| 11 | 51 | 391 | 0.152 | 0.135 | 248 |  | 0.33 | 218 | 0.68 |
|  | 41;74 | 391;570 | 0;1.05 | 0.052;0.180 | 29;391 |  | 0.17;0.51 | 462;651 | 0.38;1.10 |
| 12 | 127 | 304 | 0.562 | 0.069 | 401 |  | 0.29 | 1226 | 0.64 |
|  | 115;146 | 272;397 | 0.042;0.736 | 0.029;0.108 | 198;700 |  | 0.19;0.39 | 1201;1324 | 0.35;1.02 |
| 13 | 164 | 263 | 0.717 | 0.064 | 932 | 0.450 | 0.30 | 1226 | 1.07 |
|  | 145;192 | 248;337 | 0.583;0.914 | 0.037;0.089 | 897;1009 | 0.400;0.508 | 0.20;0.41 | 1126;1309 | 0.62;1.48 |
| 14 | 158 | 256 | 0.268 | 0.000 | 29 |  | 0.34 |  |  |
|  | 140;198 | 239;368 | 0.041;0.685 | -0.021;0.017 | 0;85 |  | 0.15;0.50 | ;518 | ;0.63 |
| 15 | 68 | 434 | 0.820 | 0.081 | 198 | 0.251 | 1.14 | 1226 | 0.83 |
|  | 55;85 | 343;665 | 0.544;1.37 | 0.009;0.134 | 80;252 | 0.187;0.329 | 0.97;1.28 | 802;1282 | 0.47;1.22 |
| 16 | 127 | 626 | 2.27 | 0.067 | 248 | 1.147 | 1.29 | 777 | 1.71 |
|  | 105;159 | 577;708 | 1.73;3.26 | 0.019;0.114 | 172;352 | 1.071;1.22 | 0.69;1.56 | 413;812 | 1.44;2.03 |
| 17 | 142 | 523 | 1.04 | 0.145 | 437 | 0.861 | 1.07 | 473 | 1.80 |
|  | 126;161 | 475;555 | 0.782;1.39 | 0.015;0.300 | 219;653 | 0.706;1.011 | 0.77;1.47 | 399;721 | 1.49;2.08 |
| 18 | 112 | 391 | 0.721 | 0.002 |  |  | 0.67 | 575 | 1.22 |
|  | 86;132 | 281;364 | 0.361;1.09 | -0.017;0.021 |  |  | 0.41;0.95 | ;602 | ;2.48 |
| 19 | 164 | 262 | 0.310 | -0.045 |  |  | 0.34 |  |  |
|  | 150;196 | 250;345 | 0.050;0.717 | -0.061;-0.024 |  |  | 0.18;0.48 | ;575 | ;0.61 |
| 20 | 177 | 256 | 0.897 | -0.024 | 85 | 0.160 | 0.34 | 1309 | 1.64 |
|  | 161;198 | 221;266 | 0.729;1.07 | -0.041;-0.006 | 0;308 | 0.131;0.193 | 0.21;0.52 | 1211;1469 | 1.07;2.62 |
