# Supplementary material for: Evaluating contribution of the cellular and humoral immune responses to the control of shedding of Mycobacterium avium spp. paratuberculosis in cattle
Source: Vet Res. 2015 Jun 19;46(1):62. doi: 10.1186/s13567-015-0204-1 (PMC4474352; doi:10.1186/s13567-015-0204-1)
Supplement: Additional file 4: — Akaike weights of the 4 different subsets of models fitted to the MAP shedding data. Models are “G” (fitting only B 0 and r), “Th1” (fitting B 0, r, k C), “Th2” (fitting B 0, r, k H), and “Th1 + Th2” (fitting B 0, r, k C, k H). The model is given in Equation 4 in the main text and details on the fitting and the definition of the best model are described in Materials and methods. Best model is highlighted in bold. [file 13567_2015_204_MOESM4_ESM.docx]

| cow ID | G | Th1 | Th2 | Th1+Th2 |
| --- | --- | --- | --- | --- |
| C01 | **0.373** | 0.336 | 0.129 | 0.162 |
| C02 | 0 | 0 | 0.006 | **0.994** |
| C03 | 0 | 0.174 | **0.767** | 0.059 |
| C04 | 0 | 0 | 0.074 | **0.926** |
| C05 | **0.3** | 0.134 | 0.203 | 0.363 |
| C06 | **0.551** | 0.191 | 0.201 | 0.057 |
| C07 | **0.415** | 0.217 | 0.293 | 0.075 |
| C08 | 0 | **0.689** | 0.001 | 0.31 |
| C09 | 0.022 | **0.663** | 0.066 | 0.25 |
| C10 | 0 | 0.388 | **0.468** | 0.143 |
| C11 | 0.002 | 0.146 | 0.008 | **0.844** |
| C12 | 0.024 | 0.036 | **0.686** | 0.254 |
| C13 | 0.001 | 0.287 | **0.516** | 0.197 |
| C14 | 0.073 | **0.434** | 0.362 | 0.131 |
| C15 | 0.033 | **0.304** | 0.019 | 0.644 |
| C16 | 0.037 | 0.017 | **0.64** | 0.306 |
| C17 | 0.031 | 0.011 | 0.023 | **0.936** |
| C18 | **0.485** | 0.169 | 0.278 | 0.068 |
| C19 | 0.134 | **0.491** | 0.047 | 0.327 |
| C20 | 0 | 0.075 | **0.896** | 0.03 |
