# Supplementary material for: Evaluating contribution of the cellular and humoral immune responses to the control of shedding of Mycobacterium avium spp. paratuberculosis in cattle
Source: Vet Res. 2015 Jun 19;46(1):62. doi: 10.1186/s13567-015-0204-1 (PMC4474352; doi:10.1186/s13567-015-0204-1)
Supplement: Supplementary file 5 [file 13567_2015_204_MOESM5_ESM.docx]

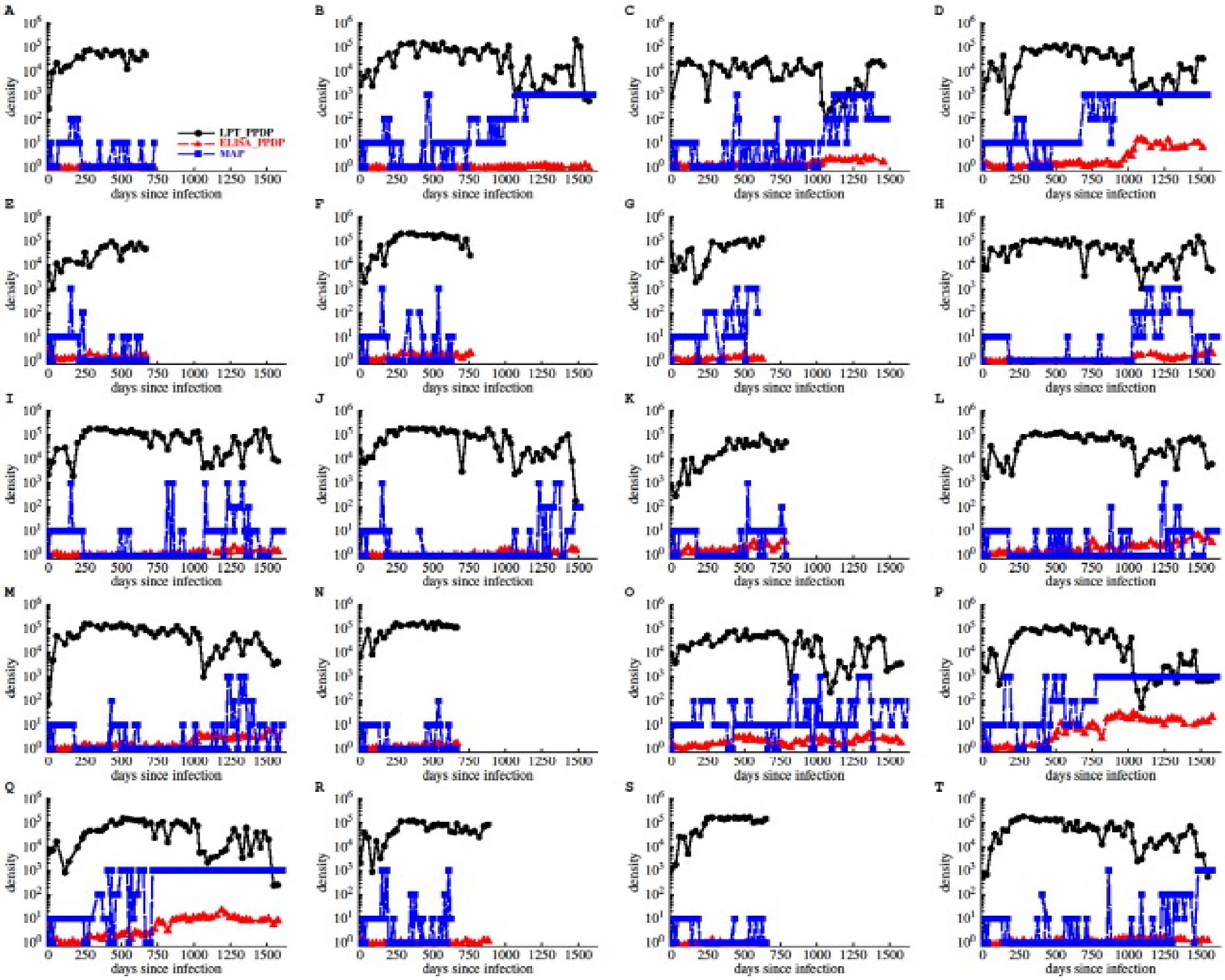

A
B
C
D
E
F
G
H
I
J
K
L
M
N
O
P
Q
R
S
T
density
days since infection
LPT_PPDP
ELISA_PPDP
MAP
