# Supplementary material for: Evaluating contribution of the cellular and humoral immune responses to the control of shedding of Mycobacterium avium spp. paratuberculosis in cattle
Source: Vet Res. 2015 Jun 19;46(1):62. doi: 10.1186/s13567-015-0204-1 (PMC4474352; doi:10.1186/s13567-015-0204-1)
Supplement: Supplementary file 6 [file 13567_2015_204_MOESM6_ESM.docx]

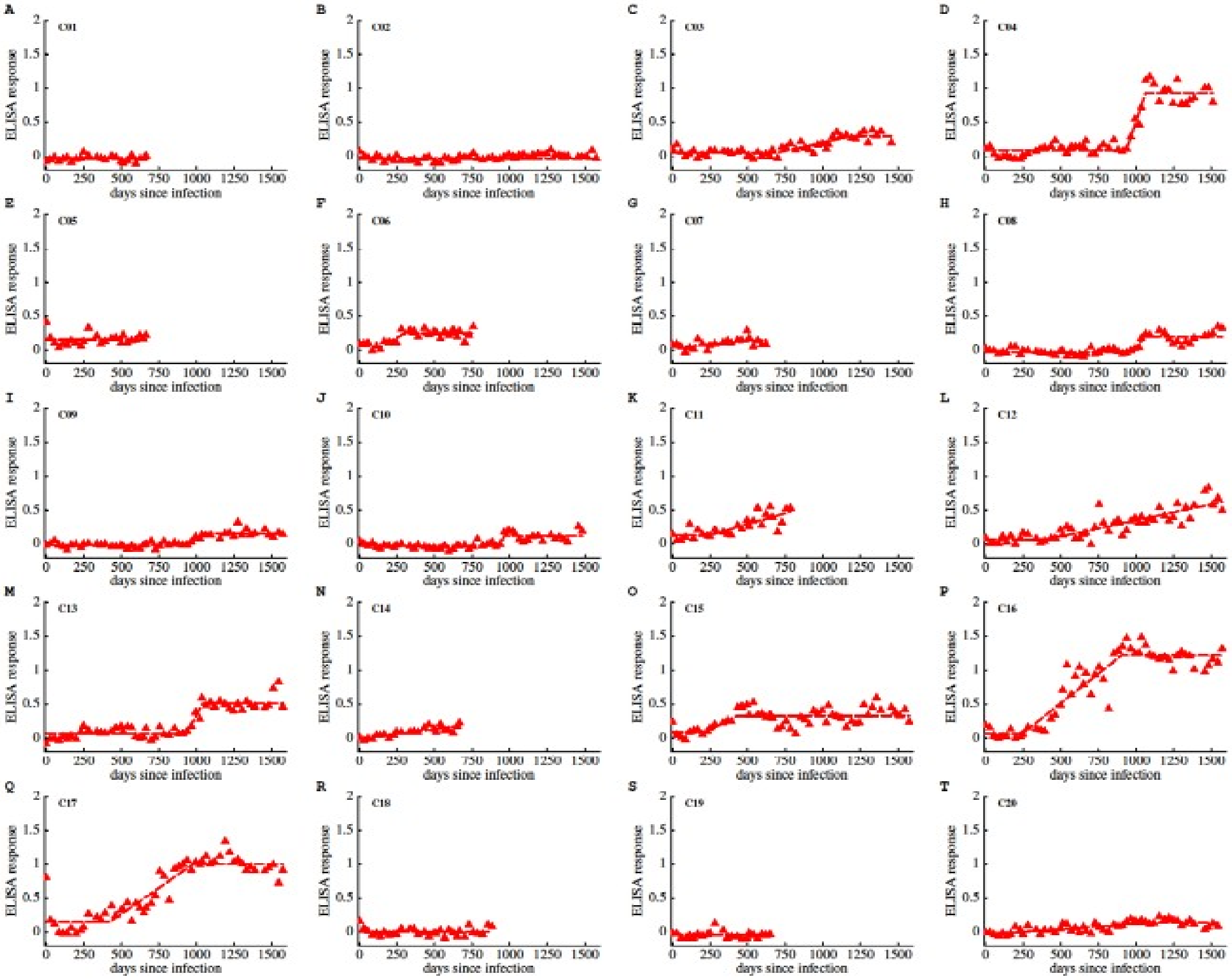

A
C01
B
C02
C
C03
D
C04
E
C05
F
C06
G
C07
H
C08
I
C09
J
C10
K
C11
L
C12
M
C13
N
C14
O
C15
P
C16
Q
C17
R
C18
S
C19
T
C20
ELISA response
days since infection
